# Supplementary material for: Effects of Resveratrol on In Vivo Ovarian Cancer Cells Implanted on the Chorioallantoic Membrane (CAM) of a Chicken Embryo Model
Source: Int J Mol Sci. 2024 Apr 16;25(8):4374. doi: 10.3390/ijms25084374 (PMC11049836; doi:10.3390/ijms25084374)
Supplement: Supplementary file 1 [file ijms-25-04374-s001.zip › ijms-2930248-supplementary/Figure S1.pdf]

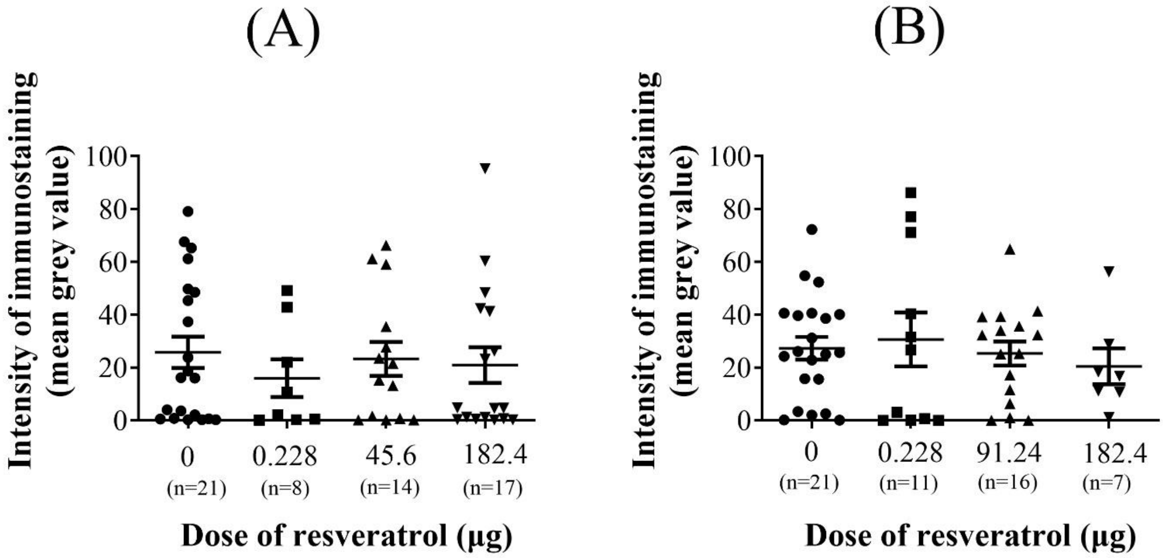

Supplementary Figure S1. Immunohistochemical staining of Ki-67 antigens in OVCAR-8 (**A**) and SKOV-3 (**B**) tumour implants treated with three doses of resveratrol (0.228 (5 µM), 45.6 (1 mM), 91.24 (2 mM), and 182.4 (4 mM) µg) for six days. The immunostaining of the antigens were quantitated using a Fiji image J software, n = number of sectioned tumour implants.
