# Supplementary material for: Effects of Resveratrol on In Vivo Ovarian Cancer Cells Implanted on the Chorioallantoic Membrane (CAM) of a Chicken Embryo Model
Source: Int J Mol Sci. 2024 Apr 16;25(8):4374. doi: 10.3390/ijms25084374 (PMC11049836; doi:10.3390/ijms25084374)
Supplement: Supplementary file 1 [file ijms-25-04374-s001.zip › ijms-2930248-supplementary/Figure S2.pdf]

Supplementary Figure S2. Western Blot membranes showing proteins extracted from OVCAR-8 and SKOV-3 tumour implants. The OVCAR-8 cell lines are represented by six control tumour implants and eight tumour implants treated with resveratrol at 91.24 µg (2 mM). Additionally, the SKOV-3 cell line data includes nine control tumour implants and eight tumour implants treated with resveratrol at 91.24 µg (2 mM), C = control, and Res = Resveratrol.

OVCAR-8 cell line

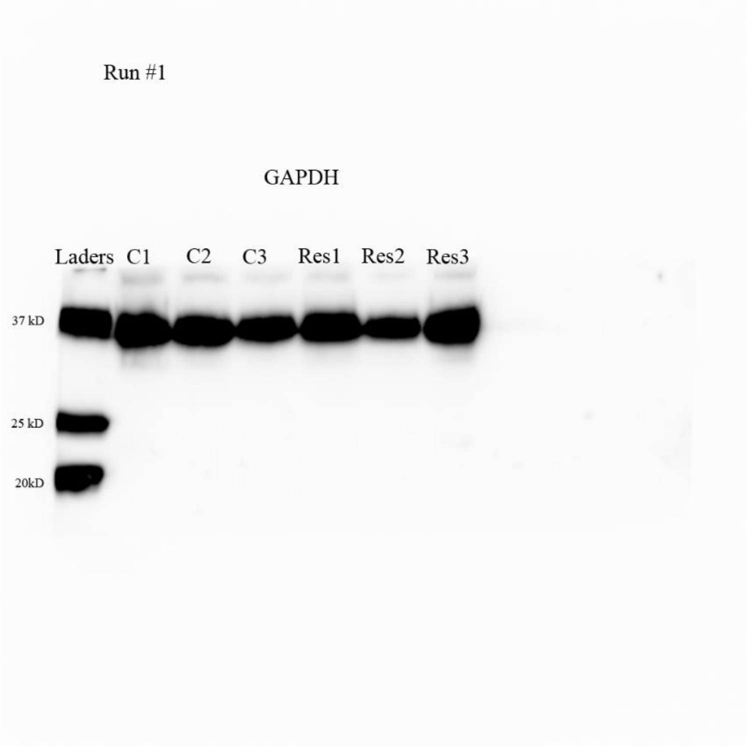

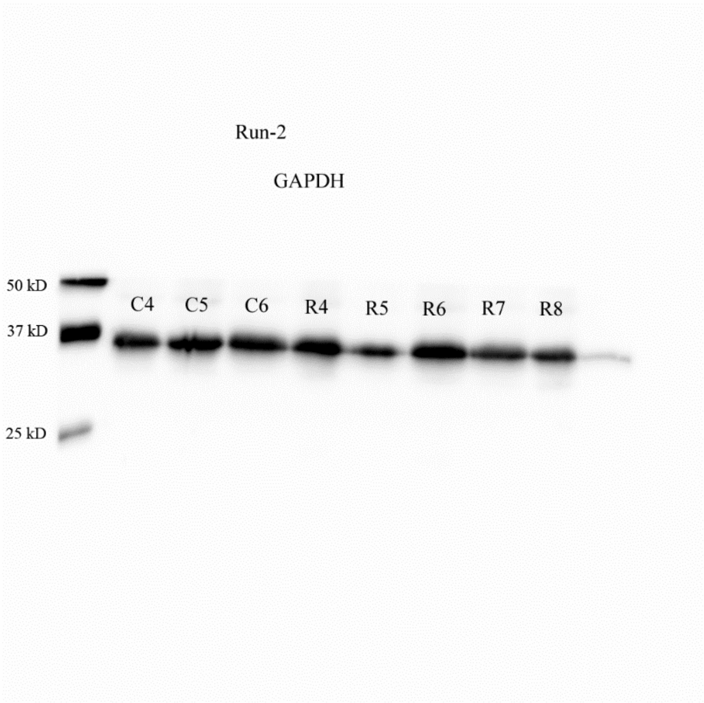

Run-1

NF-kB

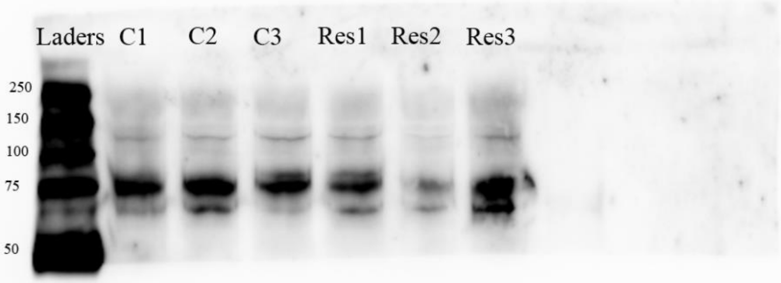

Run-2

NF-kB

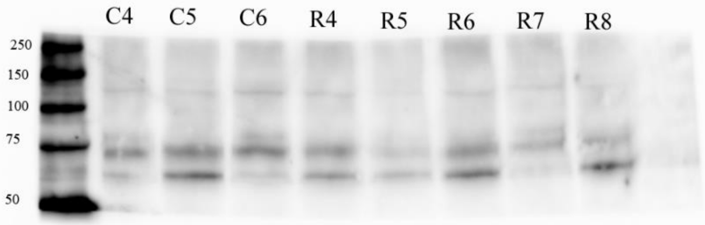

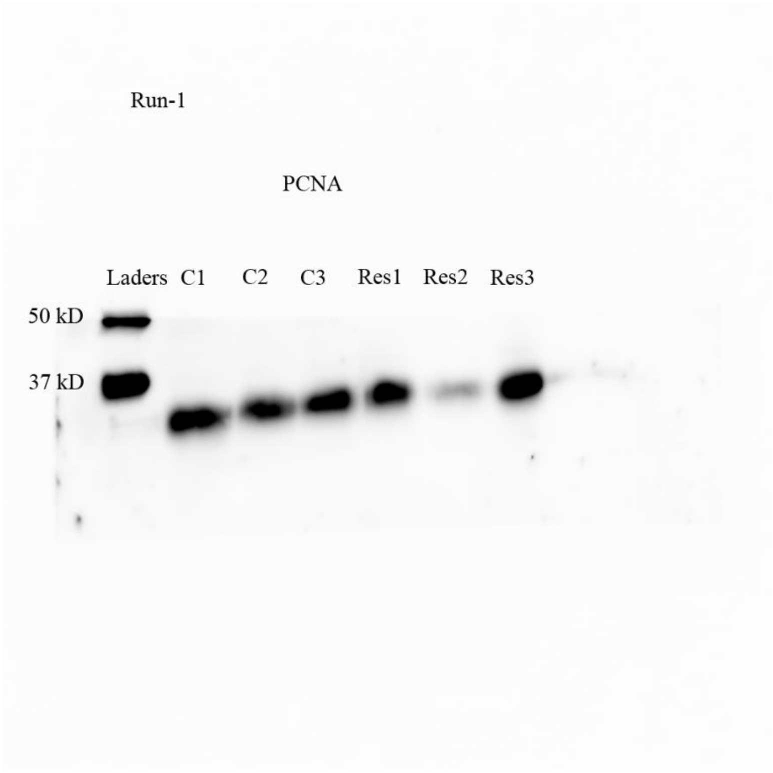
Run-1
PCNA
Laders C1 C2 C3 Res1 Res2 Res3
50 kD
37 kD

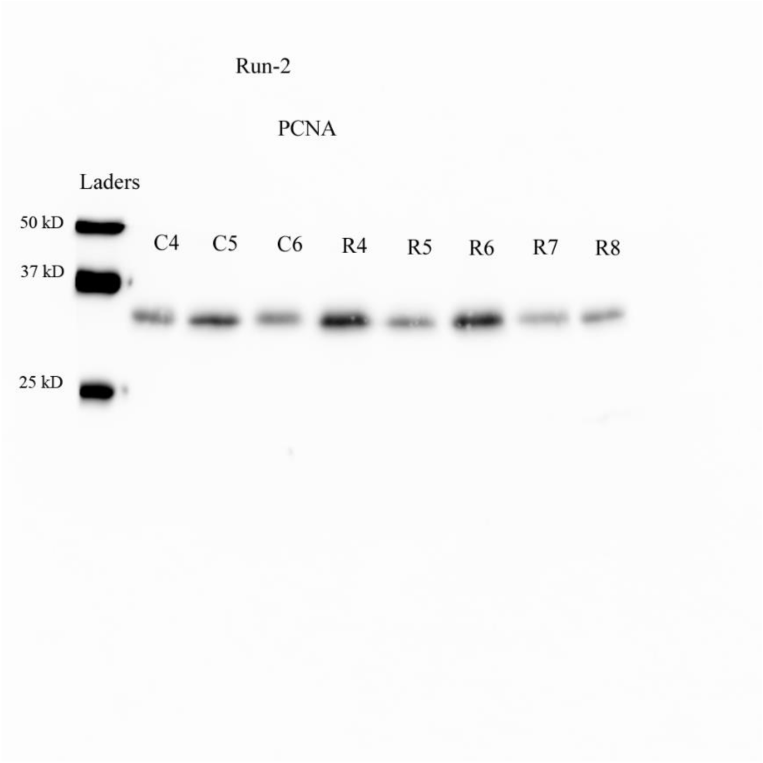
Run-2
PCNA
Laders
50 kD
37 kD
25 kD
C4 C5 C6 R4 R5 R6 R7 R8

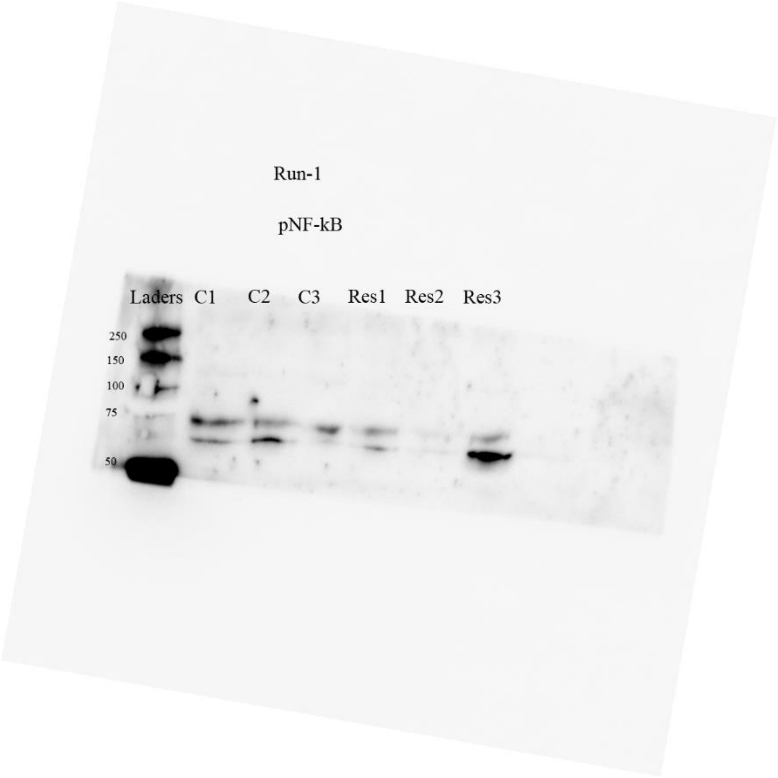
Run-1
pNF-kB
Laders C1 C2 C3 Res1 Res2 Res3
250
150
100
75
50

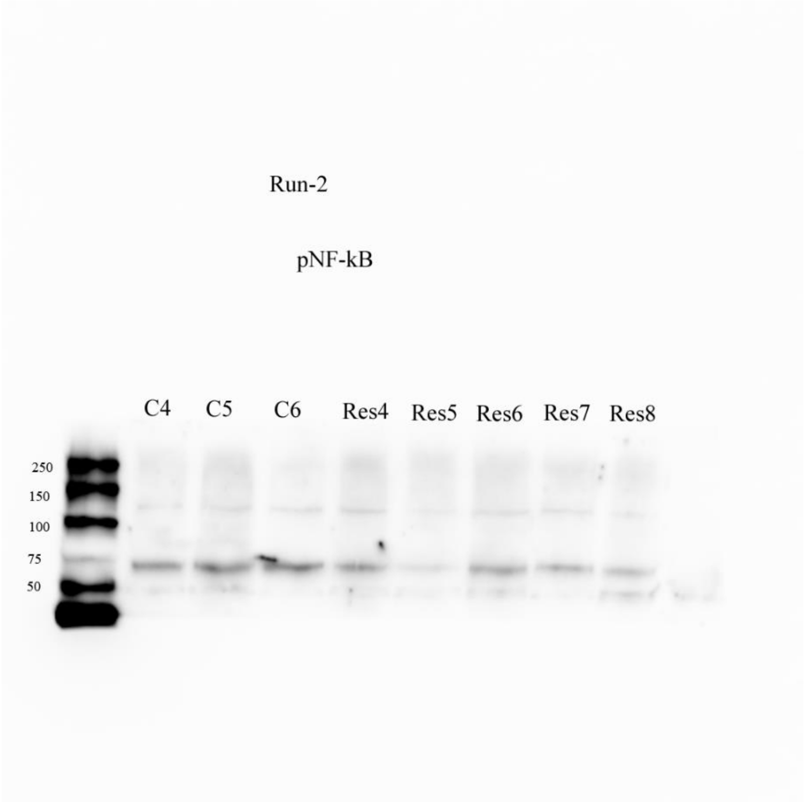
Run-2
pNF-kB
C4 C5 C6 Res4 Res5 Res6 Res7 Res8
250
150
100
75
50

Run-1

SLUG

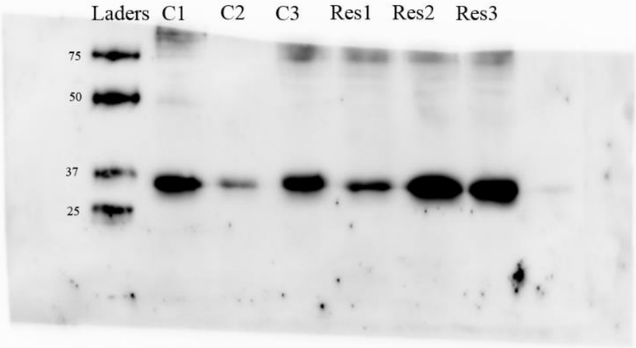

Run-2

SLUG

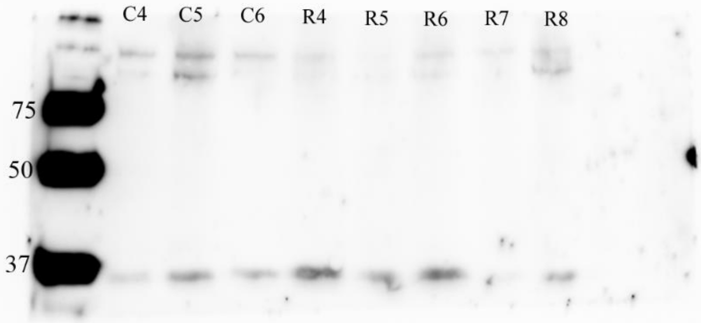

SKOV-3

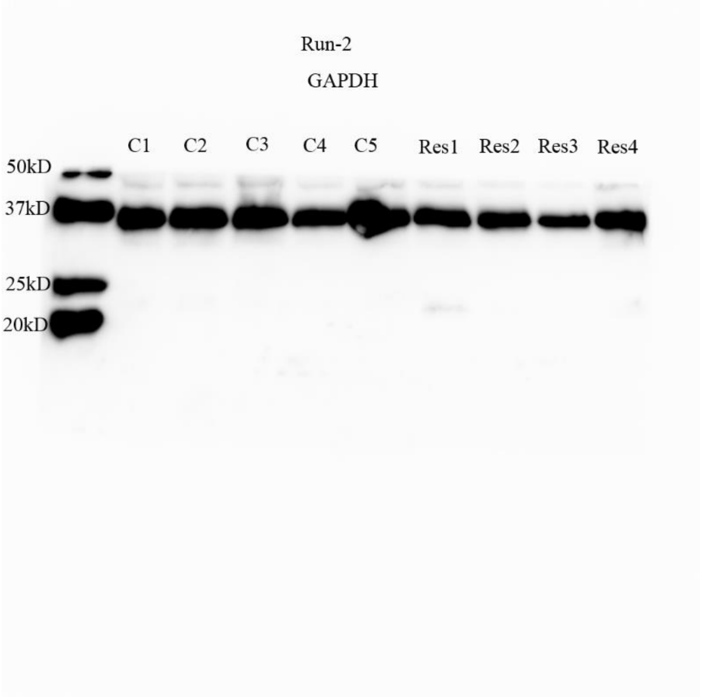
Run-2
GAPDH
C1
C2
C3
C4
C5
Res1
Res2
Res3
Res4
50kD
37kD
25kD
20kD

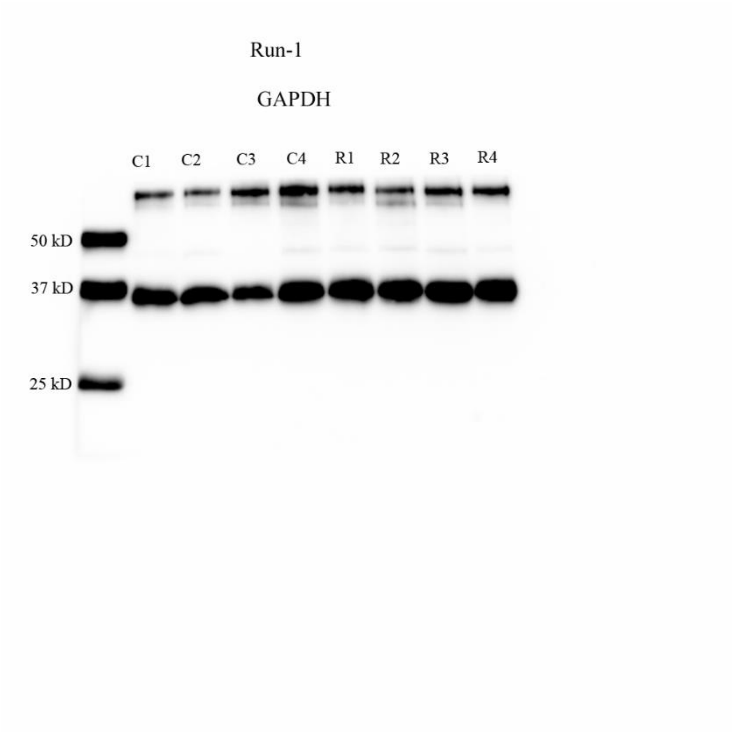
Run-1
GAPDH
C1
C2
C3
C4
R1
R2
R3
R4
50 kD
37 kD
25 kD

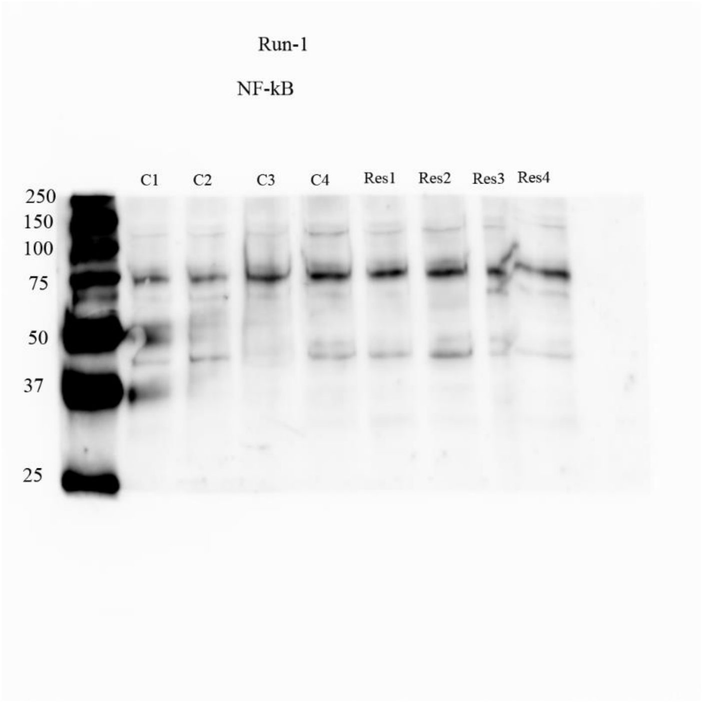
Run-1
NF-kB
C1
C2
C3
C4
Res1
Res2
Res3
Res4
250
150
100
75
50
37
25

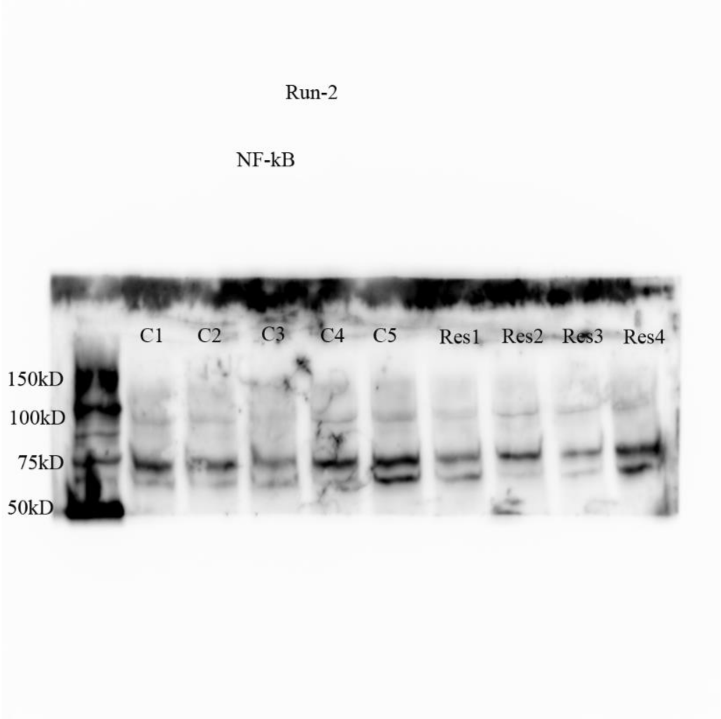
Run-2
NF-kB
C1
C2
C3
C4
C5
Res1
Res2
Res3
Res4
150kD
100kD
75kD
50kD

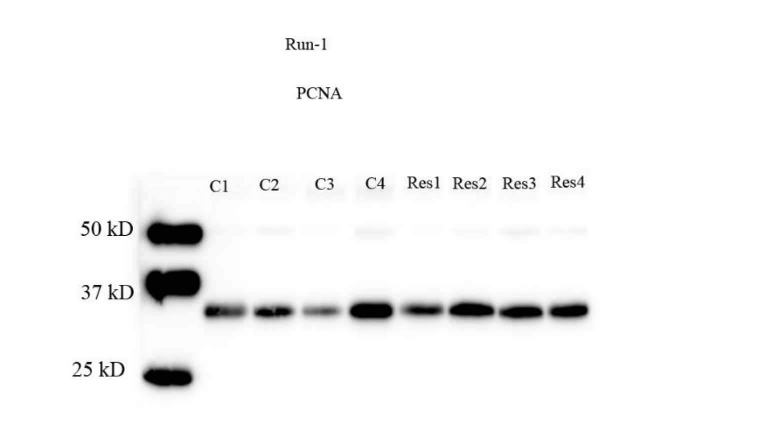
Run-1
PCNA
C1 C2 C3 C4 Res1 Res2 Res3 Res4
50 kD
37 kD
25 kD

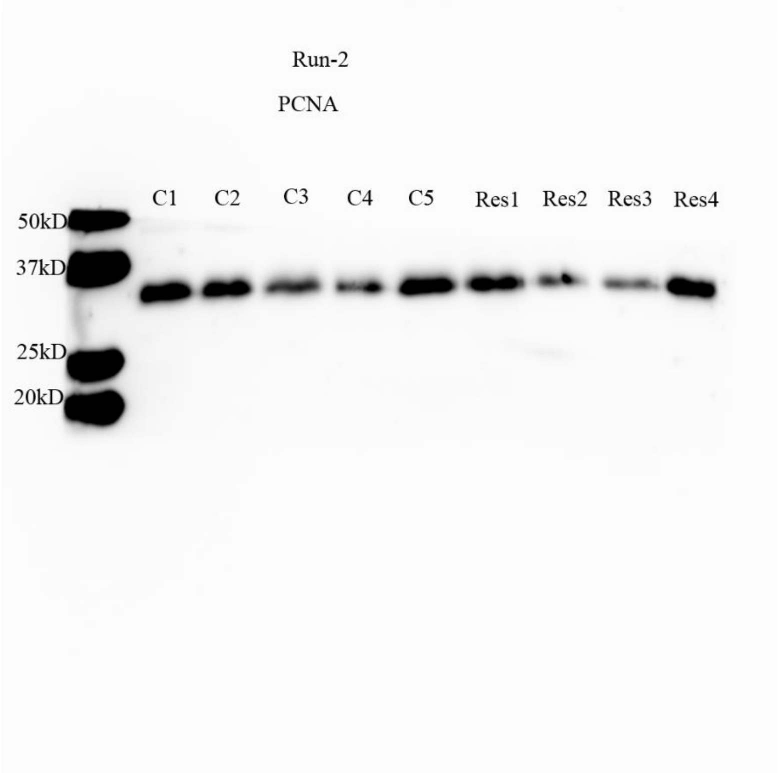
Run-2
PCNA
C1 C2 C3 C4 C5 Res1 Res2 Res3 Res4
50kD
37kD
25kD
20kD

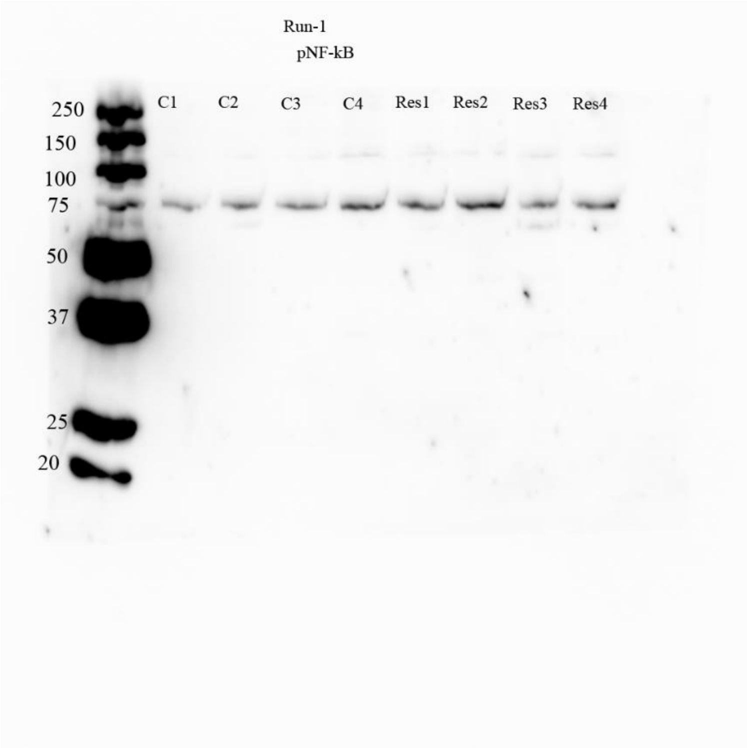
Run-1
pNF-kB
C1
C2
C3
C4
Res1
Res2
Res3
Res4
250
150
100
75
50
37
25
20

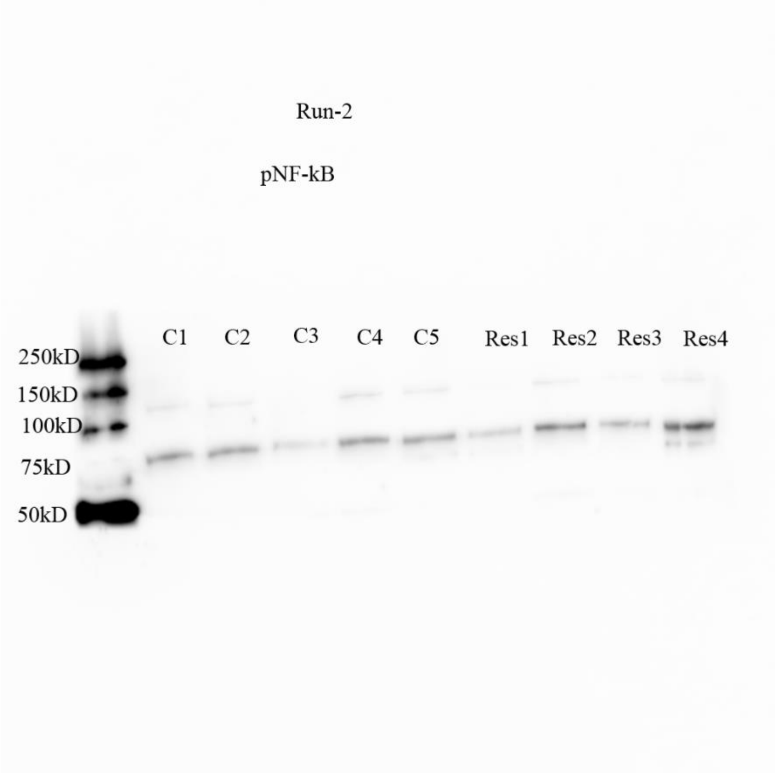
Run-2
pNF-kB
C1
C2
C3
C4
C5
Res1
Res2
Res3
Res4
250kD
150kD
100kD
75kD
50kD

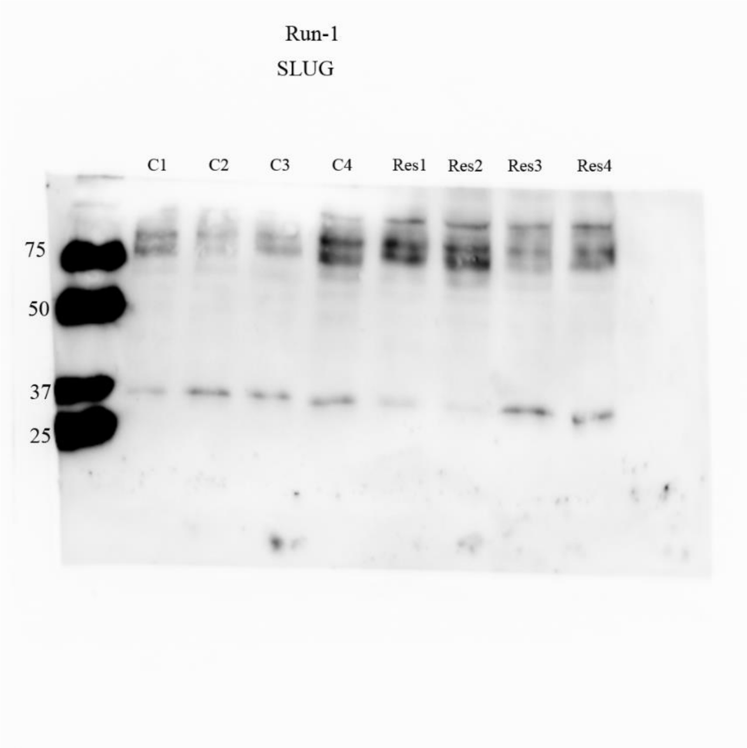
Run-1
SLUG
C1
C2
C3
C4
Res1
Res2
Res3
Res4
75
50
37
25

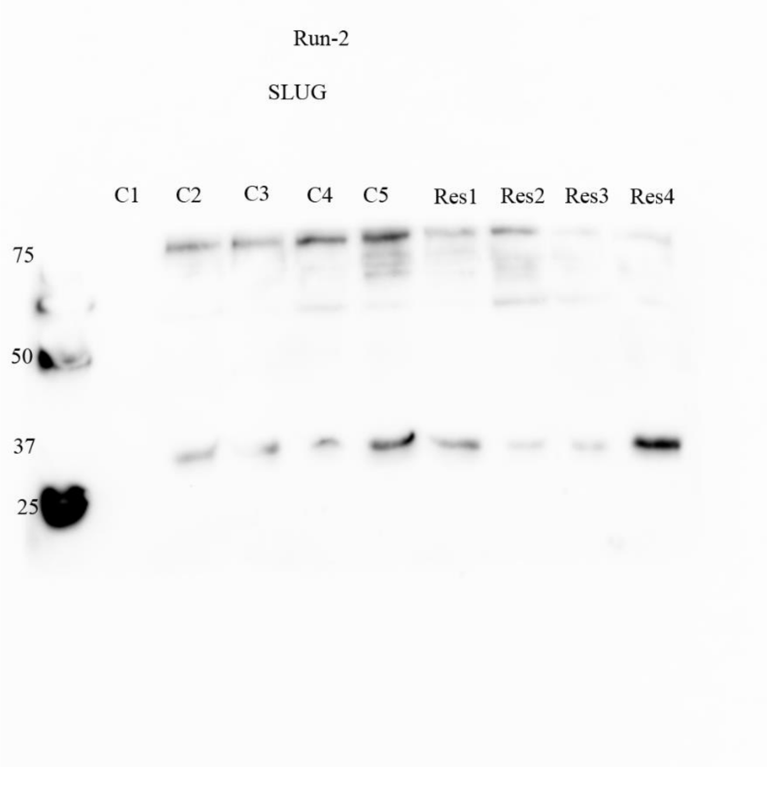
Run-2
SLUG
C1
C2
C3
C4
C5
Res1
Res2
Res3
Res4
75
50
37
25
